# Detection of Plasma EGFR Mutations in NSCLC Patients with a Validated ddPCR Lung cfDNA Assay

**DOI:** 10.7150/jca.31326

**Published:** 2019-07-10

**Authors:** Qiao-mei Guo, Lin Wang, Wen-jun Yu, Li-hua Qiao, Ming-na Zhao, Xiao-meng Hu, Ya-meng Sun, Sheng Ni, Yun-hua Xu, Jia-tao Lou

**Affiliations:** 1 Department of Laboratory Medicine, Shanghai Chest Hospital, Shanghai Jiao Tong University, Shanghai, China; 2 Shanghai Lung Cancer Center, Shanghai Chest Hospital, Shanghai Jiao Tong University, Shanghai, China; 3 Bio-Chain Biological Technology Co., Ltd, Shanghai, China

**Keywords:** cell-free DNA, droplet digital PCR, *EGFR*, NGS, non-small cell lung cancer

## Abstract

**Purpose**: The clinical utility of cell-free DNA (cfDNA) to assess *EGFR* mutations is increasing. However, there are limited studies determining their clinical validity and utility. The value of cfDNA assays in cancer management remains controversial.

**Methods**: In this study, we first evaluated the analytical performance of the ddPCR Lung cfDNA Assay. We next analyzed the concordance of the results with tissue amplification refractory mutation system PCR (ARMS-PCR) and plasma next-generation sequencing (NGS) genotyping. Finally, we assessed its clinical utility by exploring the association of cfDNA *EGFR* mutations with metastatic sites and the efficacy of EGFR-TKIs treatment.

**Results**: The ddPCR Lung cfDNA Assay demonstrated a limit of blank of 1 droplet per reaction, an analytical specificity of 100%, and detection limit of 0.05%, 0.05%, and 0.1% for* E746_A750del*, *L858R*, and *T790M*, respectively. With tissue ARMS-PCR as a standard for comparison, the clinical sensitivity and specificity of ddPCR were 62.5% (15/24) and 100% (82/82) for *E746_A750del*, and 75.0% (15/20) and 94.2% (81/86) for *L858R*, respectively. The ddPCR showed high concordance with NGS in determining cfDNA *EGFR* mutations. Patients with bone and/or brain metastasis showed a higher detection rate and mutant abundance of cfDNA *EGFR* mutations compared to those with other sites of metastasis. Moreover, EGFR-TKIs treatment was effective in patients with sensitive *EGFR* mutations in either plasma cfDNA or tumor tissue-derived DNA.

**Conclusions**: We validated in this study that the ddPCR Lung cfDNA Assay is reliable for detection of *EGFR* mutations in lung cancers, in terms of analytical performance, clinical validity and utility.

## Introduction

Lung cancer is the leading cause of cancer-related mortality worldwide. Non-small cell lung cancer (NSCLC) accounts for approximately 80% of lung cancers. The majority of the NSCLC patients are unresectable at their initial diagnosis and suffer from desperate prognosis. In the past decade, the advancement of molecular targeted therapies has greatly improved the survival of patients with advanced NSCLC, in which EGFR-tyrosine kinase inhibitors (EGFR-TKIs) was a tremendous success. Epidermal growth factor receptor (EGFR) is a transmembrane protein that plays a complicated role in signal transduction and cellular processes. *EGFR* mutations have been recently reported to occur in 10% of NSCLC patients in Western countries and 30-50% of cases in East Asia 1. The most common *EGFR* mutations are located in the tyrosine kinase domain of the *EGFR* gene, including deletions in exon 19 (45%) and a missense mutation (*L858R*) in exon 21 (40-45%) 2, 3. These alterations result in constitutive activation of the downstream signaling and serve as the driver of neoplastic transformation and progression. The presence of these mutations are associated with EGFR-TKI sensitivity and hence may serve as a predictive biomarker of response to the related targeted therapies 4, 5. Despite showing a response rate of over 70% in patients harboring *EGFR* driving mutations, the diseases eventually progress at a median time ranging from approximately 10-13 months 4, 6, 7. The most common mode of acquired resistant in *EGFR* mutation-positive patients is the development of a secondary point mutation in the *EGFR* active domain, substituting a bulky methionine amino acid for threonine (*T790M*) and inhibiting the binding of *EGFR*-TKIs. Currently, the *T790M* mutation was estimated to represent 50-60% of resistance to first- and second-generation TKIs 8-10. Hence, it is necessary to detect and monitor *EGFR* mutations throughout the treatment and surveillance of patients with advanced NSCLC.

In clinical practice, tumor tissues are insufficient for *EGFR* genotyping in at least 20% of advanced NSCLC patients for various reasons including insufficient availability of neoplastic tissue, lack of appropriate tumor tissue for biopsy, or that a biopsy is not technically feasible 11, 12. Beyond that, a single biopsy snapshot could not represent the global feature of a heterogeneous tumor and repeat biopsy for treatment monitoring is challenging. Recently, circulating tumor DNA (ctDNA) as a non-invasive “real-time” biomarker is being extensively studied, aiming to substitute tissue genotyping and provide an insight into the tumor heterogeneity. These may allow the molecular analysis in patients with tissue sample unavailable and offer a chance to monitor the mutational evolution of the tumors during treatment, possibly predicting disease progression. In fact, ctDNA has been studied in several specific areas to be an alternative surrogate for molecular analysis in cancer patients 13-15.

Nevertheless, the mutation analysis and mutant abundance quantification of the trace amounts of ctDNA still pose a challenge to the plasma DNA-based detection techniques. The commonly used methods for plasma ctDNA *EGFR* mutation analysis include amplification refractory mutation system PCR (ARMS-PCR), droplet digital PCR (ddPCR), and next-generation sequencing (NGS) 16-19. ARMS-PCR was considered to be better suited for detection of tissue samples where mutant allele fraction (MAF) was usually higher than 1%. Testing of *EGFR* mutations as part of targeted NGS panels allows various driver mutations to be analyzed simultaneously, however, the bioinformatic pipeline to call mutations from large amounts of raw sequencing data is complicated. Digital PCR now represents a precise and sensitive single-molecule counting strategy to detect extremely low levels of genetic materials. The performance of digital PCR surpasses many quantitative methods, enabling the detection of rare mutations originating from tumor cells mixed in a large background of homologous sequences 20. Researches regarding ctDNA assays by ddPCR are rapidly growing, but there is a lack of study assessing its analytical validity, clinical validity, and utility in the real-world setting.

Here, we established a lung cancer ctDNA detection platform named ddPCR Lung cfDNA Assay. We assessed the analytical and clinical performance of the platform by evaluating DNA derived from mutant cell lines at specified MAF and clinical specimens. The clinical utility of the platform was also explored by analyzing the association between *EGFR* mutation status and metastatic sites in 108 metastatic NSCLC patients. Furthermore, the efficacy of EGFR-TKIs treatment in 122 newly diagnosed patients with sensitive *EGFR* mutations was also evaluated.

## Material and Methods

### Patients

The study was approved by the Ethics Committee of Shanghai Chest Hospital, Shanghai Jiao Tong University, Shanghai, China. From June 2016 to March 2018, NSCLC patients who were subjected to peripheral blood cfDNA *EGFR* analyses at the Shanghai Chest Hospital were enrolled in this study. Healthy individuals with similar age and gender distributions who performed regular physical check-up in our hospital and were not diagnosed with any malignant diseases were recruited and served as a control group. Signed written informed consent was obtained from each participant.

### Cell lines

Three human lung cancer cell lines including PC-9, H1975, and A549 were purchased from the American Type Culture Collection. The cells were cultured at 37°C in a 5% CO_2_ humidified atmosphere with RPMI-1640 medium supplemented with 10% heat-inactivated fetal bovine serum, 50 U/mL penicillin, 50 µg/mL streptomycin, and 2 mmol/L L-glutamine (Gibco). Among them, PC-9 contains *E746_A750del* mutation, H1975 contains *L858R* and *T790M* mutations, and A549 contains wild-type *EGFR*. The genomic DNA from these cell lines were extracted for mutation analysis in our study.

### Plasma collection and DNA extraction

Blood samples were collected into two 5-mL EDTA vacutainer tubes from each participant after informed consent had been obtained. Blood samples were immediately spun into plasma with two-step centrifuges within 2 hours. Firstly, the whole blood was centrifuged at 1600g for 10 mins at 4°C, then the supernatant was centrifuged at 16000g for 10 mins at 4°C. Cell-free DNA (cfDNA) was extracted from 4 mL of plasma using QIAamp Circulating Nucleic Acid Kit (Qiagen). Genomic DNA was extracted from lung cancer cell lines using TIANamp Genomic DNA Kit (Tiangen) according to the manufacturer's instructions.

### ddPCR

ddPCR was performed using the QX200 Droplet Digital PCR system (Bio-Rad Laboratories). Samples were prepared by mixing 10 µL ddPCR Supermix for probes (No dUTP, Bio-Rad Laboratories), 2 µL ddPCR™ probe assay Kit (Bio-Rad Laboratories) which consist of forward and reverse PCR primers and FAM or HEX-labeled fluorescent probe (specific for each mutation assay), and 8 µL of template DNA in a final reaction volume of 20 μL. Droplets were generated by a QX200 droplet generator. Endpoint PCR was performed on a T100 Thermal Cycler (Bio-Rad Laboratories). Thermal cycling profile for *EGFR* mutation assay was starting with a hot start denaturation step of 10 mins at 95°C, followed by 40 cycles of: 94°C for 30 s, 70°C for 1 min. These cycles were followed by 98°C for 10 mins and then 4°C hold. Then, PCR products were loaded into the QX200 droplet reader and analyzed by QuantaSoft version 1.7.4.0917 (Bio-Rad Laboratories). For each assay: water without templates (NTC) served as a control for detecting environmental contamination; a negative control (genomic DNA from *EGFR* wild-type A549 cell line) was used to estimate the false-positive rates; and a positive control containing genomic DNA from mutant (MT) cell lines (H1975 or PC-9) was used to verify the assay performance and determine the threshold value of fluorescent signals. Poisson distribution was used to determine the concentration of mutant DNA and calculate the MAF. The MAF is equal to the number of droplets positive for mutant FAM probe / total number of positive for mutant FAM probe plus wild-type HEX probe. The measured MAF was expressed in percentage.

### NGS

Plasma ctDNA libraries were prepared with a Firefly NGS DNA Library Prep Reagent Set (AccuraGen) as described in a previous study 21. The Firefly NGS is an amplicon-based lung cancer panel targeting about 68 hotspots in *EGFR*, *KRAS*, and *BRAF* genes. The platform had a detection limit of 0.1%. All samples were sequenced using Illumina Miseq (Illumina) in a 150 bp paired-end pattern. Mutation calls data were analyzed by CometScope software (AccuraGen).

### ARMS-PCR

The paired tumor tissue DNA samples were tested by using the EGFR 29 Mutation Detection Kit (Amoy Diagnostics) according to the manufacturer's instructions. The kit detects 29 clinically-relevant *EGFR* mutations in lung cancer. Mutation status of deletions in exon 19, *L858R* and *T790M* mutations were used for the analysis in this study.

### Statistical analysis

Coincidence rates between different detection platforms were calculated by using Cohen's kappa. The average MAF between patients with bone and/or brain metastasis (B/BM) and patients with other sites of metastases (OSM) were compared by using independent sample *t*-test. Progression-free survival (PFS) was defined as the period from the date of initiation of treatment to the date of disease progression or death due to any cause. Survival curves for PFS were generated by using the Kaplan-Meier method. Log-rank tests were used to compare the survival curves among different subgroups. *P* values < 0.05 were considered to be statistically significant. All statistical analyses were performed by using SPSS software (version 24.0, SPSS Inc.) or GraphPad Prism for Windows (version 6.07, GraphPad Software).

## Results

### Patients' characteristics

**Figure 1:**
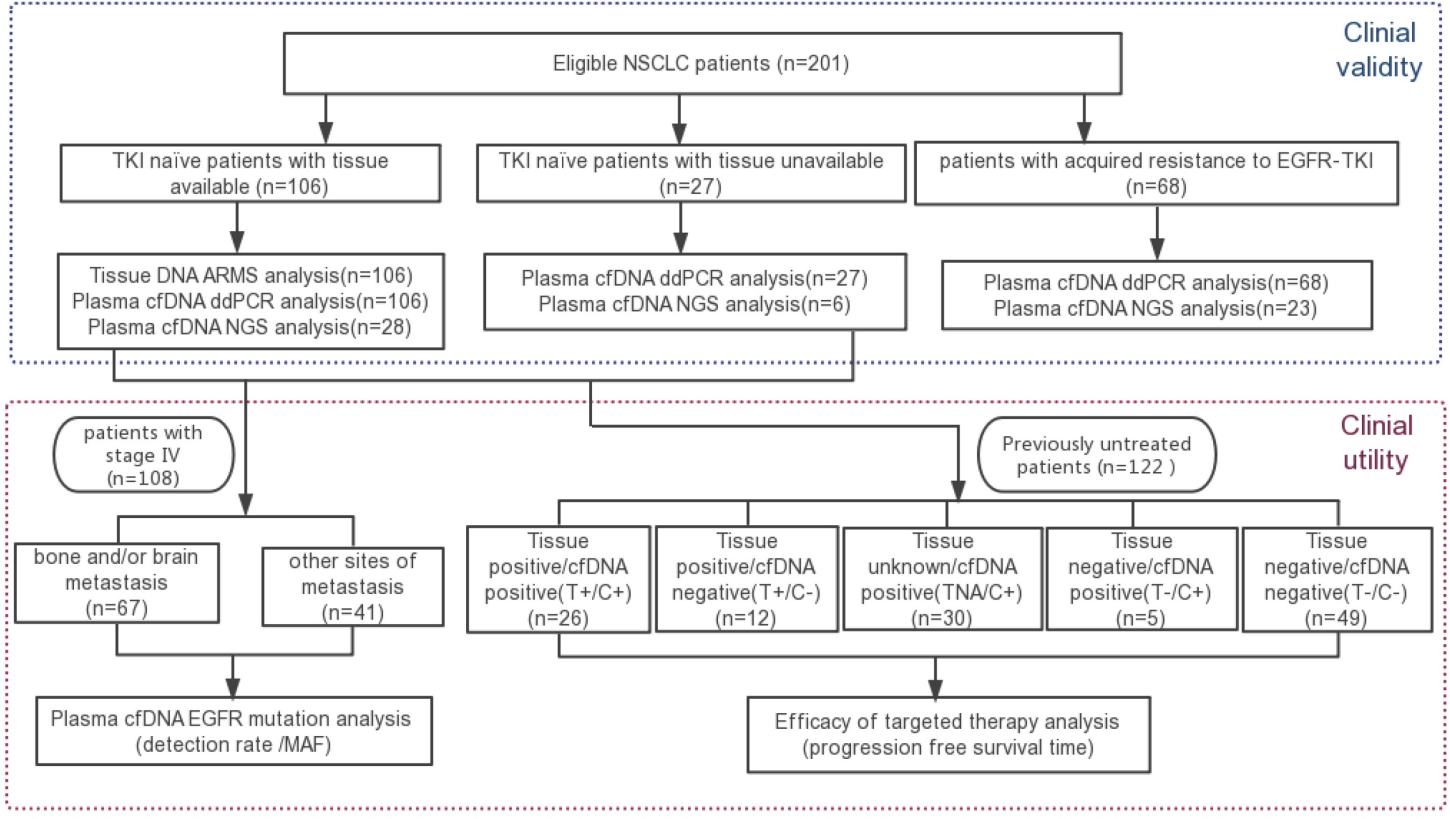
The schema for patient screening.

A total of 201 NSCLC patients were subjected to peripheral blood cfDNA *EGFR* analyses. 82.6% of patients had adenocarcinoma, 11.4% had NSCLC not otherwise specified, and 6.0% had squamous cell carcinoma. The majority of the patients were stage IV (85.6%) and only 14.4% were stage I to III. Of these, 68 patients were resistant to EGFR-TKIs, 133 patients were EGFR-TKI treatment-naïve, and 106 patients were identified retrospectively to have matched tumor tissue *EGFR* genotyping results. The schema for patient screening is shown in Figure 1, and the detailed clinical characteristics of the enrolled patients are summarised in Table 1.

### Analytical validity

**Figure 2:**
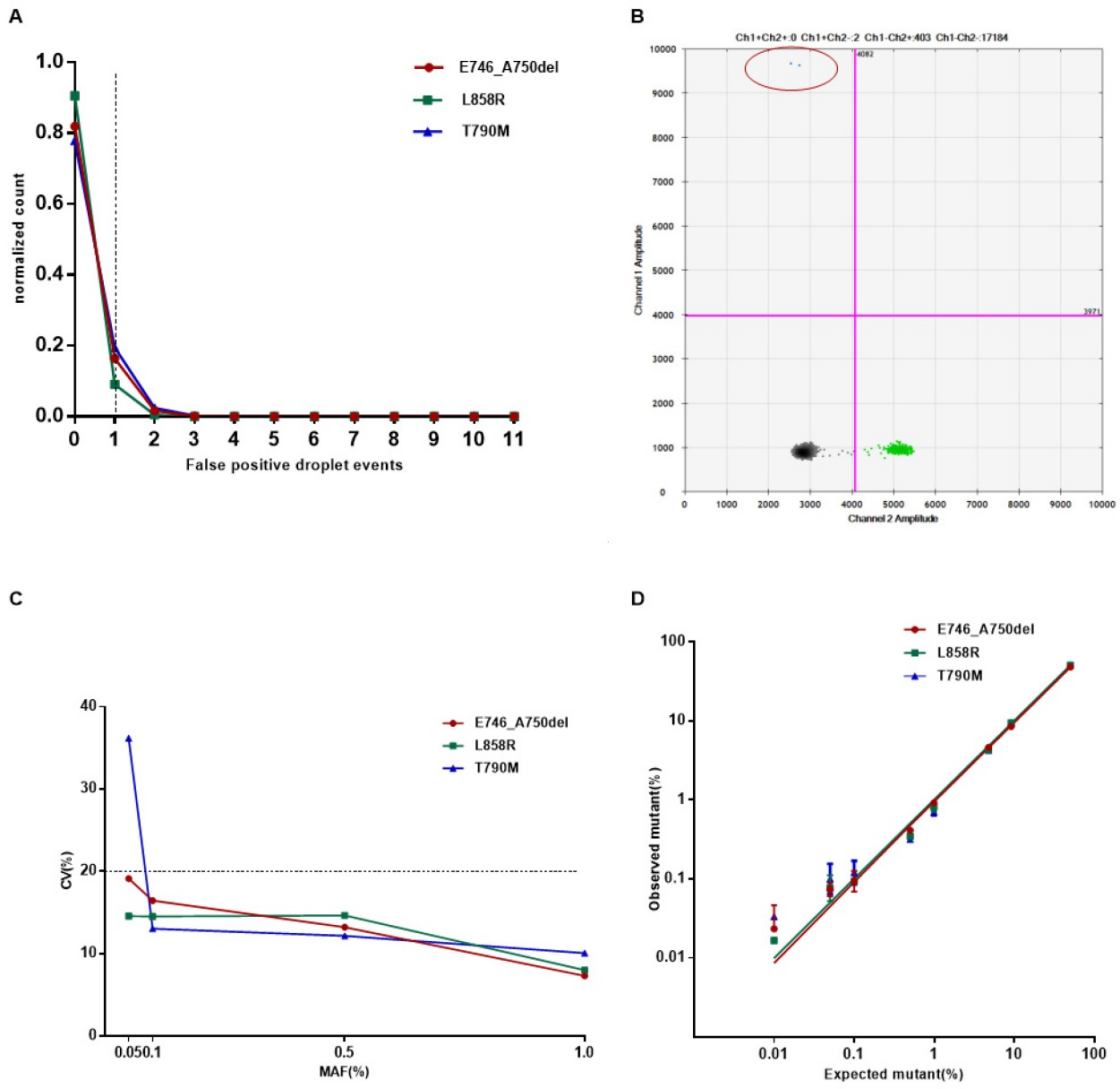
The analytical validity of the ddPCR Lung cfDNA Assay. (A) Determination of the limit of blank (LOB) of *E746_A750del*,* L858R*, and *T790M*. The LOBs were determined as 1 event per reaction for all the three types of mutation from the 95% confidence interval of the Poisson model fit. (B) The representative positive result detected by ddPCR. Droplets containing mutative targets are double positive for FAM and HEX (shown in the upper right quadrant). (C-D) The analytical sensitivity and linearity of *E746_A750del*, *L858R*, and* T790M*. The serially diluted positive cell line DNA with different MAF was duplicated 20 times for sensitivity and coefficient of variation (CV%) was calculated and exhibited. For linearity assay, the serially diluted positive cell line DNA were tested in triplicates at each concentration.

Firstly, we intended to evaluate the analytical performance of the ddPCR Lung cfDNA Assay by determining the Limit of Blank (LoB) for single variants, and defining the detection limit, analytical specificity, and linearity of the assay. By using plasma cfDNA from 20 healthy donors, the LoB was decided by fitting a Poisson model to the false-positive frequency distribution for each target and evaluating the 95% one-tailed upper limit of the model distribution as previously described 22. The number of false-positive droplet event is 1 for all of the* E746_A750del*, *L858R*, and *T790M* (Figure 2A). Therefore, samples were considered “positive” if assays showed equal to or more than 2 droplets at the expected amplitude for all the three types of mutations (Figure 2B).

Then, the detection limit was assessed by using cell line-derived genomic DNA with known variants at specified MAF of 1%, 0.5%, 0.1%, and 0.05%. A total of 50 ng of input DNA with varying proportions of mutant DNA mixed into wild-type DNA was subjected to ddPCR. To evaluate the robustness of the assay, all reactions were repeated 20 times at each MAF. The minimum MAF that our system can reliably detect (CV<20%) was considered as the detection limit. Results indicated that the detection limit was 0.05% for* E746_A750del* and *L858R*, and 0.1% for *T790M*, respectively (Figure 2C).

The analytical specificity was evaluated by testing different input amounts of wild-type genomic DNA mixed into potentially interfering substances such as bacterial genomic DNA. No false-positive events above the defined threshold of 2 droplets per reaction were observed for each type of mutation. In addition, the three positive DNA templates were not cross-reacting or interfering with each other (Table 2). In summary, the analytical specificity of the ddPCR assay was 100%.

To determine the linearity, cell line-derived genomic DNA containing the desired *EGFR* mutations was serially diluted with wild-type DNA to obtain samples with MAF at 50%, 10%, 5%, 1%, 0.5%, 0.1%, 0.05%, and 0.01%. All reactions for each MAF were performed in triplicates. On average, all three types of mutations showed good linearity (R^2^>0.99, Figure 2D) and wide dynamic range (from 1:20000 to 2000:20000 copies).

### Clinical validity

The clinical validity of ddPCR Lung cfDNA Assay was first evaluated in terms of the concordance of *EGFR* mutations detected between paired tumor tissue specimens and plasma samples obtained from 106 EGFR-TKIs treatment-naïve patients. The median amount of input cfDNA per reaction was 5.66 ng (range: 2.21-76 ng). With tissue genotyping results as a standard for comparison, the sensitivity and specificity of ddPCR were 62.5% (15/24) and 100% (82/82) for *E746_A750del*, and 75.0% (15/20) and 94.2% (81/86) for *L858R*, respectively (Table 3).

In view of the biological discrepancies between plasma and tissue samples, we next compared our ddPCR Lung cfDNA Assay with a commercial NGS platform for the detection of *EGFR* mutations in cfDNA from 57 patients. The concordance rates between the two platforms were 89.5% (51/57) for *E746_A750del*, 98.3% (56/57) for *L858R*, and 93.0% (53/57) for *T790M*, respectively (Table 4). Furthermore, ddPCR Lung cfDNA Assay showed a 42.7% (29/68) positive rate for *T790M* in cases with acquired resistance to TKI, which is consistent with previous findings of ASTRIS and AURA 17 studies 17, 23, 24.

### Clinical Utility

#### Correlation of plasma *EGFR* mutations with metastases

**Figure 3:**
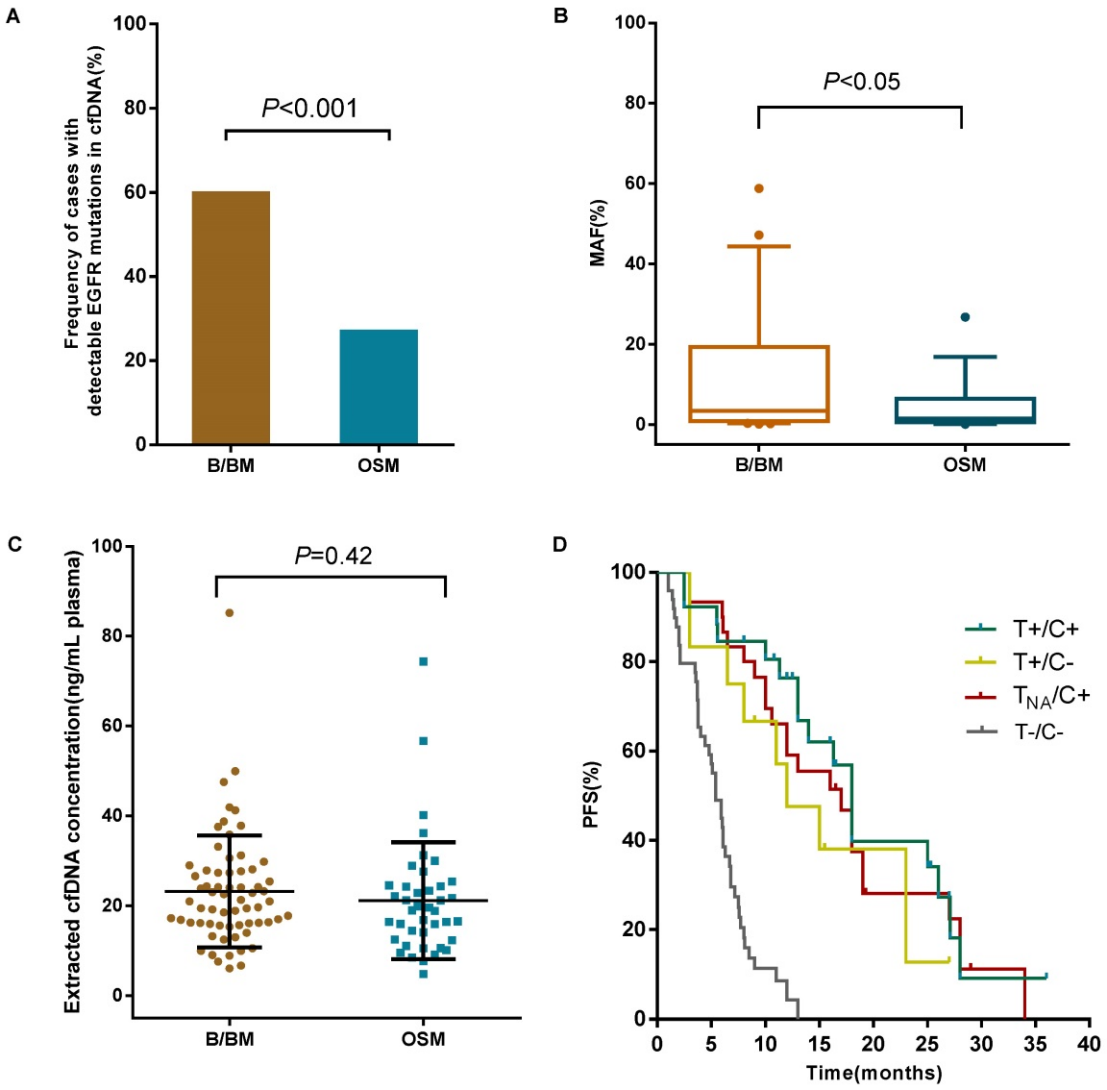
Clinical utility of cfDNA* EGFR* mutation detection by the ddPCR assay. (A) The frequency of cases with detectable *EGFR* mutations in cfDNA between B/BM group and OSM group was presented. (B) The mutant allele fraction (MAF) in patients with B/BM and OSM. The average percentage of MAF in plasma *EGFR* is shown by a midline (outliers excluded). (C) The extracted cfDNA concentration between B/BM group and OSM group. The mean concentration of cfDNA was analyzed in 67 B/BM patients and 41 OSM patients. (D) Survival curves of progression-free survival (PFS) in 122 newly diagnosed NSCLC patients.

B/BM are common metastatic sites in patients with lung cancer. The prognosis of these patients is poor with a median survival of less than 1 year 25. We retrospectively investigated the relationship between metastases and *EGFR* mutation status in cfDNA from 108 TKI-naïve patients with stage IV NSCLC. For this cohort of patients, 59.7% (40 out of 67 patients who developed B/BM) had detectable sensitive *EGFR* mutations in cfDNA, in contrast, only 26.8% (11/41) with OSM had detectable sensitive *EGFR* mutations in cfDNA (*P*<0.001, Figure 3A). Then, we analyzed whether *EGFR* mutant abundance in plasma was correlated with the occurrence of B/BM. In 51 patients with sensitive* EGFR* mutations in cfDNA, the average MAF in patients with B/BM was 9.9%, which was obviously higher than that in patients with OSM (3.5%, *P*<0.05, Figure 3B), even though the extracted cfDNA concentration was similar between the two groups (23.3 ng/mL vs 21.2 ng/mL, *P*=0.42, Figure 3C). Moreover, patients who developed brain metastases harbored higher *EGFR* mutant abundance with an average MAF of 18.2%, whereas patients who developed only bone metastases carried slightly lower *EGFR* mutant abundance with an average MAF of 6.6% (*P*<0.05). Interestingly, 8 out of 11 patients (72.7%) with brain metastases accompanied by bone metastases. The above results indicated that B/BM in lung cancer patients was associated with positive *EGFR* mutations in cfDNA and that the higher abundance of sensitive *EGFR* mutations in cfDNA might be a risk factor for developing brain metastases.

#### Prognostic significance of plasma *EGFR* mutations detected by ddPCR

To emphasize the clinical significance of different *EGFR* mutation status, we analyzed the progression-free survival (PFS) in 122 newly diagnosed patients treated with EGFR-TKIs or standard chemotherapy. Patients were stratified into subgroups based on the genotyping result: Group A, Tissue-positive/cfDNA-positive *EGFR* mutation (T+/C+); Group B, Tissue-positive/ctDNA-negative *EGFR* mutation (T+/C-); Group C, Tissue-unknown/ctDNA-positive *EGFR* mutation (T_NA_/C+); Group D, Tissue-negative/ctDNA-negative *EGFR* mutation (T-/C-); and Group E, Tissue-negative/ctDNA-positive *EGFR* mutation (T-/C+) . The PFS of each group was investigated (Figure 3D). Patients with *EGFR* mutations in either tissue or cfDNA (Group A-C) had a significantly improved PFS (13 months, n=68) compared to patients harbored wild-type *EGFR* in both tissue and cfDNA (Group D: 5.4 months, n=49, *P*<0.001). The median PFS of Group A was 15 months, which was slightly longer than that of Group B and Group C, (11.5 and 13 months, respectively), however, no statistical difference was reached (*P*=0.202). Furthermore, we observed the two of five patients with Tissue-negative /cfDNA-positive EGFR mutation (T-/C+, group E) showed a PFS of 8 and 14 months, respectively. These results suggest that *EGFR* mutation status in either tissue and cfDNA was associated with clinical response to TKIs. *EGFR* analysis in cfDNA is a potential alternative method for those patients who cannot obtain sufficient tumor tissue sample. Plasma cfDNA-based* EGFR* mutations analysis by ddPCR is useful in guiding clinical decisions in patients with insufficient or unavailable tumor specimens.

## Discussion

Plasma cfDNA genotyping has already been utilized in guiding clinical decision-making in advanced NSCLC patients. The joint assessment from experts of American Society of Clinical Oncology and the College of American Pathologists has declared that there is insufficient evidence of analytical validity, clinical validity, and utility for the majority of ctDNA assays in cancer 26. We, therefore, established the ddPCR Lung cfDNA Assay and assessed its performance according to the suggestions. Our results showed that the detection limit of the ddPCR Lung cfDNA Assay was 0.05% for *E746_A750del* and *L858R*, and 0.1% for* T790M*, respectively. Using the defined 2 events per reaction as the threshold, the analytical specificity was 100% for all three types of *EGFR* mutations.

In a real-world setting, the practical sensitivity of the ddPCR assay is strongly dependent on the amount of available DNA sample 27, 28. To yield sufficient input DNA for ddPCR assay, we optimized the pre-analytical specimen processing. A sequential pair of centrifugations of 4 mL peripheral anticoagulant venous blood was performed within 2 hours after collection 26. The median amount of cfDNA is 5.22 ng per reaction and 95% of the samples are over 3 ng (equivalent to about 1000 copies of the genome), which will generate a theoretical sensitivity of 0.1%. Zhang, et al. demonstrated that the sensitivity of ddPCR was more than 60% for the cases with cfDNA inputs of 2-5 ng per reaction when tumor-tissue *EGFR* mutation served as a standard for comparison 27. Our results were in agreement with their findings.

The ddPCR Lung cfDNA Assay exhibited a high concordance with tissue ARMS-PCR result for the detection of *E746_A750del* and *L858R*, which was consistent with the previously reported concordance rate ranging from 70% to 90% 13, 21, 29. However, the clinical sensitivity of the ddPCR Lung cfDNA Assay for detecting *E746_A750del* (62.5%, 15/24) was modest. The false-negative results may be arise from the different coverage of the platforms, the heterogeneity of tumor, the complexity of cfDNA shedding, and other clinical factors. Particularly, the tissue ARMS-PCR assay used in the present study covered multiple types of exon 19 deletion mutations, not just *E746_A750del*. This could be the critical reason for the decrease in sensitivity of detecting plasma *E746_A750del* by the assay. In addition, we found that 29.0% (11/38) of patients who have discordant *EGFR* mutations performed their tumor tissue ARMS-PCR detection at least one months earlier than plasma ddPCR detection, suggesting that the discrepancies may be caused by molecular evolution of tumors.

For cfDNA *T790M* mutation detection, the ddPCR Lung cfDNA assay shows a high concordance with a well-developed commercial NGS platform, proving the reliability of the cfDNA Lung ddPCR Assay in *T790M* mutation detection. Moreover, in the present study, the positive rate of plasma *T790M* in patients with acquired resistance to EGFR-TKIs was 42.7%, which was consistent with the previously reported detection rate. Several famous studies based on the NSCLC population in the Asia-Pacific, namely ASTRIS and AURA 17, had also reported similar positive rates of plasma *T790M* with our current study, ranging from 36.1% to 56%. The difference in the *T790M* detection rates may be due to clinical factors such as chemotherapy, smoking, different types of primary mutation, targeted therapy, the duration of systemic treatment, and etc. 9, 30-32.

For clinical utility, we found that patients with B/BM demonstrated a higher detection rate of sensitive *EGFR* mutations in plasma cfDNA than those with OSM. Patients with B/BM carried a higher abundance of sensitive *EGFR* mutations in cfDNA. Subgroup analysis revealed that MAF in patients with brain metastasis was obviously higher than that in patients with only bone metastasis and that in patients with OSM. In addition, most of the brain metastasis patients were accompanied by bone metastasis. Survival analysis revealed that patients with either Tissue-positive or cfDNA-positive* EGFR* mutation had a longer PFS after EGFR-TKIs treatment compared to those with wild-type *EGFR* mutations receiving conventional standard treatments, suggesting that the combined use of tissue and cfDNA assays may be a better strategy to select patients eligible for EGFR-TKIs treatment in a real-world clinical setting.

Our study has several limitations. First, self-made materials were used to establish the analytical sensitivity of the ddPCR platform instead of commercial cfDNA reference which might be more reliable. Second, the sample size is too small in the subgroup of patients with Tissue-negative /cfDNA-positive EGFR mutation (T-/C+). Several studies also reported the occurrence of T-/C+ patients 33-36, however, they accounted for only 4.0% of the study population (155 in 3834 cases). The efficacy of EGFR-TKIs to those patients was also observed as reported by Mok, et al. 37. Besides, the sensitivity of ddPCR is highly depended on the input amount of DNA template. Enrichment or pre-amplification is required before ddPCR assay for low concentration samples. Despite these limitations, our study is valuable in terms of the well-validated analytical performance and clinical utility in real-world setting.

In conclusion, we have developed the ddPCR Lung cfDNA Assay for detecting *EGFR* mutations with both high sensitivity and specificity, and with the robustness needed in clinical practice. The assay could be valuable in the detection of actionable *EGFR* mutations in patients who are unable to undergo repeat biopsies and possibly detecting “missed” mutations by standard tissue genotyping due to tumor heterogeneity. Furthermore, this approach would be particularly useful in predicting the B/BM in NSCLC patients.

## Figures and Tables

**Table 1:** Clinical characteristics of the 201 enrolled advanced NSCLC patients.

Characteristic		Parameter value
**Gender; No. (%)**		
	Male	96 (47.8)
	Female	105 (52.2)
**Age, years; means (range)**		61.5 (23-87)
**Histology; No. (%)**		
	Adenocarcinoma (AC)	166 (82.6)
	Squamous cell carcinoma (SCC)	12 (6.0)
	NSCLC not otherwise specified	23 (11.4)
**Stage; No. (%)**		
	Ⅰ-Ⅲ Ⅳ	29 (14.4) 172 (85.6)
**Tissue mutation status; No.**		
	19dels	24
	L858R	20
	Wild type	62
	Unavailable	95
**Prior treatment status; No.**		
	Acquired resistant to EGFR-TKIs	68
	EGFR-TKIs naïve patients	133
**Metastasis in TKI-naïve patients; No.**		
	Bone and/or brain (B/BM)	67
	Other sites of metastasis (OSM) Without metastasis	41 25

**Table 2:** The analytical specificity of the ddPCR assay.

Template	Mutant allele fraction (mutant/wild-type)		
	Ex19del	T790M	L858R
Low concentration of wild-type genomic DNA	0/1780	0/1840	0/1900
Medium concentration of wild-type genomic DNA	0/7400	3.6/7720 (0 event)	0/7960
High concentration of wild-type genomic DNA	0/13780	1.8/13800 (0 event)	0/1442
*L858R*and *T790M* positive genomic DNA	0/11720	-	-
*E746_A750del* positive genome DNA	-	5.6/20120 (1event)	0/2038
DNA from *Staphylococcus aureus*	0/0	0/0	0/0
DNA from *Escherichia coli*	0/0	0/1.8	0/2

**Table 3:** Performance of the ddPCR assay for detecting *EGFR* mutations in comparison with Tissue ARMS-PCR genotyping.

	cfDNA ddPCR	Tissue ARMS-PCR		Performance of ddPCR		
		Positive	Negative	Sensitivity (%)	Specifity (%)	Concordance (%)
***E746_A750del***	**Positive**	15	0	62.5	100	91.5
	**Negative**	9	82			
***L858R***	**Positive**	15	5	75.0	94.2	90.6
	**Negative**	5	81			

**Table 4:** Performance of the ddPCR assay for detecting cfDNA *EGFR* mutations in comparison with NGS.

	cfDNA ddPCR	NGS		Concordance	Kappa	*P*
		Positive	Negative	(%)		
***E746_A750del***	**Positive**	16	3	89.5		
	**Negative**	3	35		0.763	<0.001
***L858R***	**Positive**	14	1	98.3		
	**Negative**	0	42		0.954	<0.001
***T790M***	**Positive**	6	2			
	**Negative**	2	47	93.0	0.709	<0.001

## Abbreviations

*EGFR*: epidermal growth factor receptor
ddPCR: droplet digital polymerase chain reaction
NSCLC: non-small cell lung cancer
EGFR-TKIs: epidermal growth factor receptor tyrosine kinase inhibitors
ctDNA: circulating tumor DNA
cfDNA: cell-free DNA
LOB: limit of blank
ARMS: amplification refractory mutation system
NGS: next-generation sequencing
MAF: mutant allele fraction
PFS: progression-free survival
